# Surface proteome of plasma extracellular vesicles as mechanistic and clinical biomarkers for malaria

**DOI:** 10.1007/s15010-023-02022-x

**Published:** 2023-03-24

**Authors:** Anna Lena Jung, Malene Møller Jørgensen, Rikke Bæk, Marie Artho, Kathrin Griss, Maria Han, Wilhelm Bertrams, Timm Greulich, Rembert Koczulla, Stefan Hippenstiel, Dominik Heider, Norbert Suttorp, Bernd Schmeck

**Affiliations:** 1 Institute for Lung Research, Universities of Giessen and Marburg Lung Center, Philipps-University Marburg, German Center for Lung Research (DZL), Marburg, Germany; 2 Core Facility Flow Cytometry - Bacterial Vesicles, Philipps-University Marburg, Marburg, Germany; 3 Department of Clinical Medicine, Aalborg University Hospital, Aalborg, Denmark; 4 Department of Clinical Immunology, Aalborg University Hospital, Aalborg, Denmark; 5 Department of Mathematics and Computer Science, Philipps-University Marburg, Marburg, Germany; 6 Department of Internal Medicine/Infectious Diseases and Respiratory Medicine, Charité-Universitätsmedizin Berlin, corporate member of Freie Universität Berlin, Humboldt-Universität zu Berlin, and Berlin Institute of Health, Berlin, Germany; 7 Medizinische Klinik m.S. Hämatologie und Onkologie, Charité-Universitätsmedizin Berlin, Berlin, Germany; 8 Department of Medicine, Pulmonary and Critical Care Medicine, University Medical Center Giessen and Marburg, Philipps-University Marburg, Marburg, Germany; 9 Center for Synthetic Microbiology (Synmikro), Philipps-University Marburg, Marburg, Germany; 10 Member of the German Center for Infectious Disease Research (DZIF), Marburg, Germany

**Keywords:** Malaria, Extracellular vesicles, Biomarker, IFN-γ, Ensemble feature selection

## Abstract

**Purpose:**

Malaria is a life-threatening mosquito-borne disease caused by *Plasmodium* parasites, mainly in tropical and subtropical countries. *Plasmodium falciparum* (*P. falciparum)* is the most prevalent cause on the African continent and responsible for most malaria-related deaths globally. Important medical needs are biomarkers for disease severity or disease outcome. A potential source of easily accessible biomarkers are blood-borne small extracellular vesicles (sEVs).

**Methods:**

We performed an EV Array to find proteins on plasma sEVs that are differentially expressed in malaria patients. Plasma samples from 21 healthy subjects and 15 malaria patients were analyzed. The EV array contained 40 antibodies to capture sEVs, which were then visualized with a cocktail of biotin-conjugated CD9, CD63, and CD81 antibodies.

**Results:**

We detected significant differences in the protein decoration of sEVs between healthy subjects and malaria patients. We found CD106 to be the best discrimination marker based on receiver operating characteristic (ROC) analysis with an area under the curve of > 0.974. Additional ensemble feature selection revealed CD106, Osteopontin, CD81, major histocompatibility complex class II DR (HLA-DR), and heparin binding EGF like growth factor (HBEGF) together with thrombocytes to be a feature panel for discrimination between healthy and malaria. TNF-R-II correlated with HLA-A/B/C as well as CD9 with CD81, whereas Osteopontin negatively correlated with CD81 and CD9. Pathway analysis linked the herein identified proteins to IFN-γ signaling.

**Conclusion:**

sEV-associated proteins can discriminate between healthy individuals and malaria patients and are candidates for future predictive biomarkers.

**Trial registration:**

The trial was registered in the Deutsches Register Klinischer Studien (DRKS-ID: DRKS00012518).

**Supplementary Information:**

The online version contains supplementary material available at 10.1007/s15010-023-02022-x.

## Introduction

Malaria is a life-threatening disease and a major health issue in tropical and subtropical countries with 247 million cases of malaria in 2021, mainly in children under 5 years of age. *Plasmodium* parasites are transferred by mosquitos. *Plasmodium falciparum* (*P. falciparum*) is the most prevalent causative agent on the African continent and is responsible for 99.7% of malaria-related deaths globally [1]. In Africa, mortality remains high because of limited access to treatment in rural areas, making malaria a disease of poverty and developing countries. Yet, the clinical outcome of malaria infection depends on many factors including the parasite, host, geographical, and sociological factors [2].

*Plasmodium falciparum* infects red blood cells (RBCs) and causes their adherence to the lining of small blood vessels, hampering tissue perfusion [3]. Infected individuals are often dehydrated and relatively hypovolemic, which potentially exacerbates microvasculature obstruction by reduction of the perfusion pressure. Moreover, the destruction of RBCs is an inevitable part of malaria, leading to anemia and a further reduction of oxygen supply [4]. Severe malaria is a syndrome that affects several tissues and organs, including the brain [2]. One important pathophysiological feature of malaria, besides severe anemia, is lactic acidosis [5]. It is a determinant of survival and may be associated with respiratory distress syndrome [6].

*Plasmodium*-infected RBCs (iRBCs) are described to release small extracellular vesicles (sEVs) containing a variety of molecules mediating pathogenesis and intercellular communication between host cells and between host and parasite [7]. sEVs can be found in all body fluids and are released from most cell types [8]. The released cargo (nucleic acids, lipids, and proteins) depends not only on the releasing cell type, but also on cellular status, and therefore, sEVs can function as biomarkers [9]. EVs can be taken up by recipient cells where they modulate cellular functions [10]. It is known that EVs from iRBCs can be potential inducers of systemic inflammation by activating macrophages [11]. Apart from the iRBCs and *Plasmodium* itself, there might be a release of EVs from other cell types into the bloodstream, making them easily-accessible biomarkers for malaria, organ dysfunction, and even disease progression, as their acquisition only requires minimally invasive procedures [12].

Here, we analyzed the surface proteome of sEVs in the blood plasma of malaria patients in comparison to healthy controls by EV array and analyzed whether these sEV surface proteins might be suitable biomarkers for disease severity.

## Methods

### Patient samples

Patients suffering from malaria were recruited within 24 h after hospitalization and before the beginning of treatment. Inclusion criteria of patients in this analysis comprised acute illness after traveling in areas endemic for malaria and positive microscopy for *P. falciparum* infection (Giemsa stain). The patients included in this study did not use antimalarial prophylaxis. Patients with specific immunosuppressive therapy, as well as pregnant or HIV-positive patients, were excluded from the study. Additionally, healthy subjects were recruited. Blood plasma was isolated by centrifugation (3000×*g*, 10 min at room temperature) of one collected Vacutainer^®^ EDTA-tube. After centrifugation, the plasma phase was transferred and stored at -80 °C. Patients underwent routine laboratory testing, healthy volunteers only underwent a blood count.

### EV array

The microarray was produced and performed as previously described [13]. In short, the protein microarrays were produced on epoxy-coated slides and antibodies were printed on a SpotBot^®^ Extreme Protein Edition Microarray Printer with a 946MP4 pin (ArrayIt Corporation, CA, USA), as described [14]. As a positive control 100 µg/mL biotinylated human IgG was used and PBS with 5% glycerol served as a negative control. Printed slides dried overnight at ambient temperature. Anti-human antibodies used for capturing were ITGAL (HI111; Ab Biotec, CA, USA); EGFR (Antibodies-online.com, DE); CD146 (P1H12), Flotillin-1, HBEGF (4G10), HLA-DR (L243), Hsp90 (IGF1), nucleophosmin (FC82291), Tsg101 (Abcam, GB); osteopontin, SFTPD (VIF11; Acris Antibodies GmbH, DE); CD16 (3G8), CD28 (L293; BD Biosciences, CA, USA); Alix (3A9), CD63, HLA-A/B/C (W6/32), HLA-DR (HL-40; Biolegend, CA USA); ICAM-1 (R6.5; eBiosciences, MA, USA); CD9, CD81, CTLA4 (ANC152.2/8H5; LifeSpan Biosciences, WA, USA); SP-A (6F10; Novus Biologicals, CO, USA); Annexin V, CD106 (HAE-2Z), CD142 (323514), CD4 (34930), CD45 (2D1), CD80 (37711), LAMP2 (H4A3), MIC-A/B (159207), TNF-R-I, TNF-R-II, Tspan8 (45811; R&D Systems, MN, USA); AREG (S-13), Coilin (F-7), HoxA7 (743C1A), TLR3 (TLR3.7; Santa Cruz Bio, TX, USA); PD-L1 (Sino Biological Inc, China); HLA-DR/DP/DQ (HB-145; Loke Diagnostics Aps, DK); CD62E (ThermoFisher Scientific, MA, USA). All antibodies were diluted in PBS with 5% glycerol and printed in triplicates at 200 µg/mL.

### Catching and visualization of sEVs

The EV Array analysis was performed as described by Jørgensen *et al*. 2013 [15]. In short, after blocking of the microarray slides, 10 µL human plasma (diluted 1:10 in wash buffer (PBS with 0.2% Tween^®^20)) was added. Samples were incubated for 2 h at ambient temperature followed by an overnight incubation at 4 °C. The slides were then washed and incubated with a cocktail of biotinylated detection antibodies (anti human-CD9, CD63, and CD81, LifeSpan BioSciences). Detection antibodies were diluted 1:1500 in wash buffer. Cy5-labelled streptavidin (1:1500 diluted in wash buffer; ThermoFischer Scientific) was used for spot visualization. After 30 min incubation, slides were washed in wash buffer and then in ultrapure water. The dried slides were then scanned as described [14].

### Data analysis

GraphPad Prism version 6 (GraphPad software, Inc., CA, USA) and R version 3.5.1 were used for statistical analysis. Background correction of EV Array data was performed on the mean signal of triplicates and before subsequent analysis, antibody signal values were log2 transformed. An unpaired *t* test with Welch’s correction was performed to assess statistical significance. *p* values < 0.05 were considered significant. Pathway and protein–protein interaction network analyses were performed with STRING version 11.0.

### Ensemble feature selection

Importance analysis of the sEV surface proteins and ranking of features was performed using the web-interface for ensemble feature selection (EFS; http://efs.heiderlab.de) [16, 17]. EFS combines eight feature selection methods and returns the normalized ensemble importance of each parameter. These are normalized quantifications of the predictive capabilities of the given variables for classification. They are aggregated from the underlying feature selection algorithms and combined into a normalized representation for each variable and thereby reduce the bias of each single estimation.

### Ethics approval

All procedures performed in studies involving human participants were in accordance with the ethical standards of the institutional and/or national research committee and with the 1964 Helsinki Declaration and its later amendments or comparable ethical standards. The BioInflame study was approved by the ethics committee of the Charité-Universitätsmedizin Berlin (EA2/030/09) and the University Medical Center Marburg (55/17). All blood donors were at least 18 years of age and provided written informed consent for use of their blood samples for scientific purposes.

## Results

### Differential abundance of sEV surface proteins

We analyzed the plasma sEV surface protein composition and compared *P. falciparum*-infected malaria patients (*n* = 15) and healthy donors (*n* = 21). Patient characteristics are given in Table 1 and Figure S1a-c. Patients in the control group were 41.6 years and 39.7 in the malaria group. The control group comprised 38% men, whereas the malaria cohort consisted of 80% men (Table 1). Malaria patients had significantly reduced thrombocyte and neutrophil counts, while the number of monocytes was significantly increased compared to healthy controls (Figure S1a). As thrombocytopenia is a well-established diagnostic marker for malaria [18], we performed receiver operating characteristics (ROC) analysis for the cohort and thrombocytes had an area under the curve (AUC) of 0.9817 (*p* < 0.0001; Figure S1b). The parasite density ranged from 0.1–5%. In the group suffering from malaria, creatinine averaged 0.98 mg/dL, bilirubin 0.78 mg/dL, LDH 246 U/L and CRP 8.4 mg/L (Figure S1c). Malaria patients were returning from travelling to Ghana (four patients), Cameroon (three patients), Gambia, Kenya, Sierra Leone, Nigeria or Cambodia (one patient each).

**Table 1:** Patient characteristics

	Control group (*N* = 21)	Malaria (*N* = 15)
Mean age [years ± SD]	41.6 ± 11.4	39.7 ± 14.6
Gender m/f (%)	8/13 (38.1/61.9)	12/3 (80/20)

*SD* standard deviation, *m* male, *f* female

**Fig. 1:**
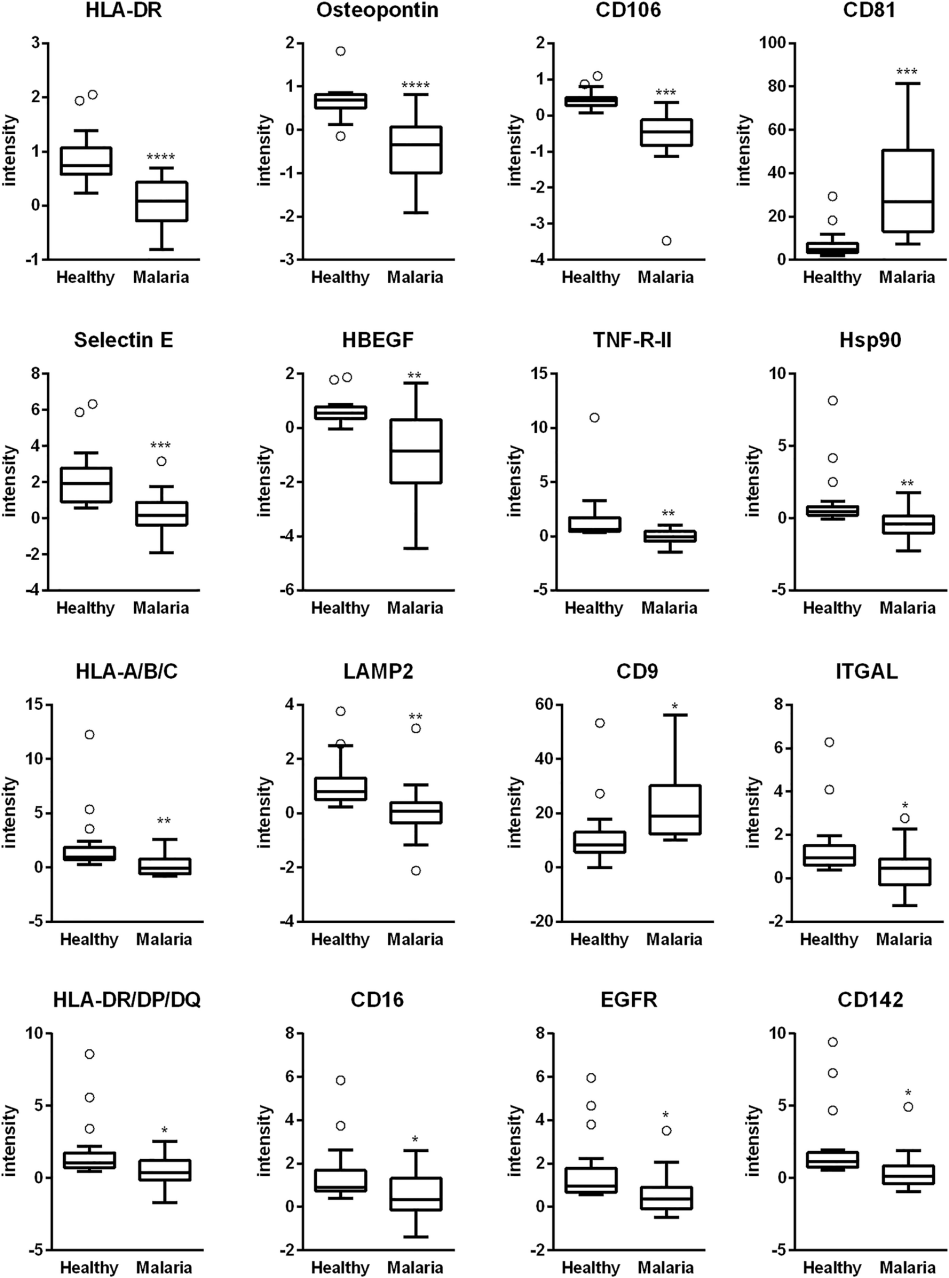
Differential abundance of sEV surface proteins. EV Array signal intensities for all significantly differentially expressed sEV surface proteins. Values were log2-transformed and are presented in Box–Whisker plots. Line is showing the median, boxes are showing 25th to 75th percentile of the data with Whiskers presenting the 1.5IQR (inter-quartile range; Tukey) and outliers. Statistics: unpaired *t* test with Welch’s correction; * in comparison to healthy; ****p < 0.0001, ***p < 0.001, **p < 0.01, *p < 0.05

The individual statistical analysis of all 40 analyzed sEV surface proteins revealed 16 of them to be differentially expressed between malaria and healthy controls (Fig. 1, Figure S2). Top candidates for the discrimination based on p-value were HLA-DR, Osteopontin, CD106, Selectin E and CD81. CD81 and CD9 were more abundant on plasma sEVs of malaria patients in comparison to healthy controls, while all other 14 proteins were significantly less abundant. As a positive control, IgG antibodies were spotted in the EV Array and we obtained similar signal intensities for healthy controls and malaria patients (Figure S1d). As the detection was as well performed with the cocktail of CD9/CD63/CD81 antibodies, we can conclude that the plasma samples of two groups contained comparable amounts of sEV.

As our malaria group contained 80% male patients, we wanted to preclude that significant differences of sEV surface proteins were only due to an unbalanced gender ratio in our malaria group. To this end, we separated male and female samples and tested for significant differences here. We only detected significant differences for HBEGF and Osteopontin (Figure S3), which were also significantly regulated between healthy and malaria. As the male group contained many diseased individuals and showed a high standard deviation in this analysis, we additionally separated healthy and malaria samples and compared female healthy to malaria and male healthy to malaria. This resulted in no significant difference for HBEGF and Osteopontin based on gender, indicating that HBEGF and Osteopontin are indeed regulated based on malaria and not on gender.

### Markers for malaria diagnosis

**Fig. 2:**
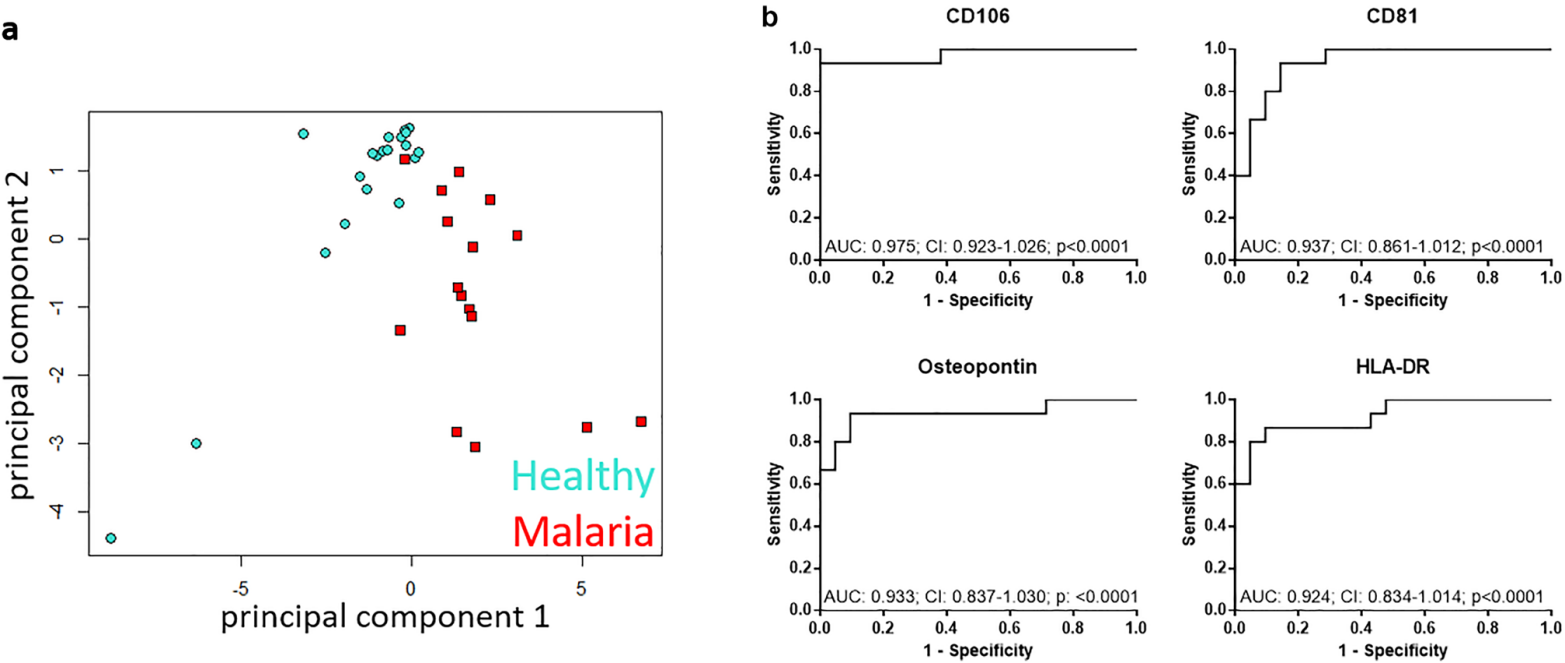
sEV surface proteins can discriminate between malaria and healthy. **a** Principal component analysis (PCA) was performed with significantly regulated sEV surface proteins. Data were scaled and centered. **b** ROC curves for discrimination between malaria and healthy are shown for CD106, CD81, Osteopontin and HLA-DR. AUC, 95% confidence interval (CI) and *p* values are given in the graphs

For the general comparison of sEVs surface protein composition between healthy and malaria, principal component analysis (PCA) was performed and revealed that the differentially expressed plasma sEV surface proteins found in Fig. 1 can be used to separate malaria patients and healthy controls (Fig. 2a). To elucidate the diagnostic value of the differentially expressed sEV surface proteins in malaria plasma, receiver operating characteristics (ROC) analysis was performed. Best AUC values were obtained for CD106 (AUC: 0.975, *p* < 0.0001), CD81 (AUC: 0.937, *p* < 0.0001), Osteopontin (AUC: 0.933, *p* < 0.0001) and HLA-DR (AUC: 0.924, *p* < 0.0001; Fig. 2b), which also were the top candidates based on significance found in Fig. 1.

**Fig. 3:**
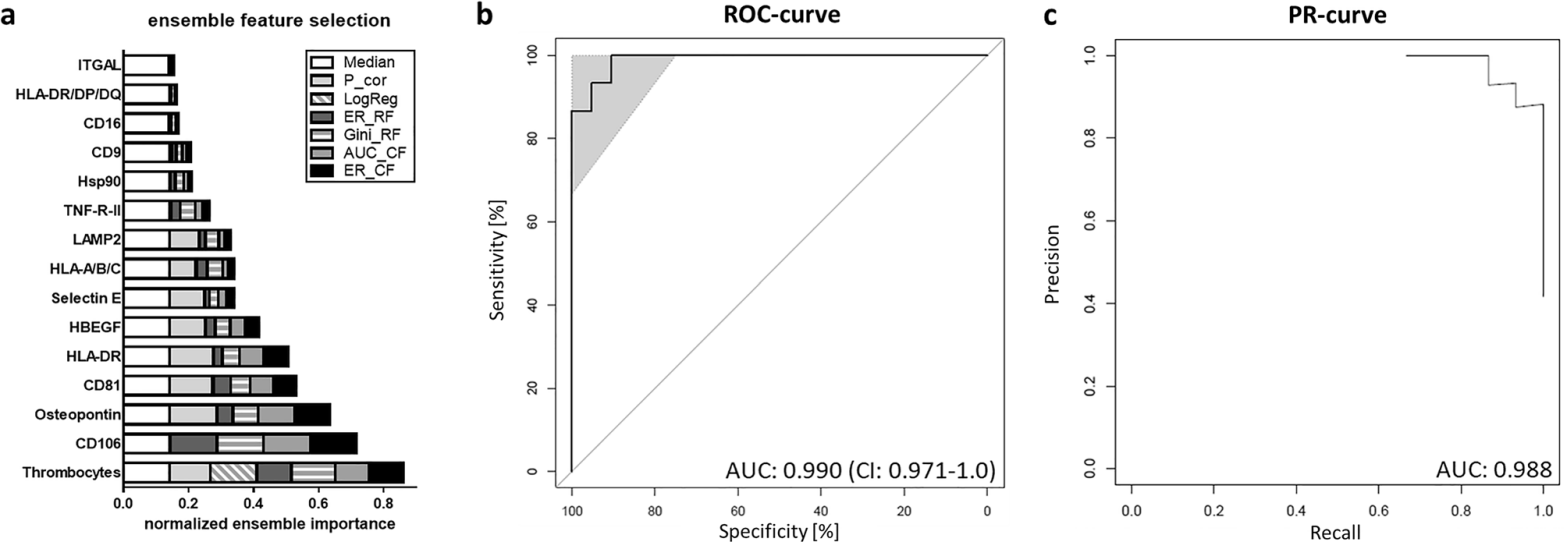
Ensemble feature selection (EFS) for healthy and malaria for all significantly regulated sEV proteins and thrombocytes. **a** Cumulative barplot of individual features for all feature selection methods is shown. Selected features are thrombocytes, CD106, Osteopontin, CD81, HLA-DR and HBEGF. P_cor: Pearson product moment correlation, LogReg: logistic regression, ER_RF: error-rate-based variable importance measure embedded in *randomForest*, Gini_RF: Gini-index-based variable importance measure embedded in *randomForest*, AUC_CF: area under the curve embedded in *cforest*, ER_CF: error-rate-based variable importance measure embedded in cf*orest*. **b**, **c** Logistic regression analysis followed by leave-one-out validation was performed. Comparison of healthy control and malaria with selected features from a. ROC analysis (**b**) and precision and recall (PR)-curve (**c**) are shown. AUC values and confidence intervals (CI) are depicted

As the diagnostic potential of a sum of sEV surface proteins might be even higher than single proteins or parameters alone, we applied the machine learning tool Ensemble Feature Selection (EFS) that combines eight feature selection algorithms and thereby improves the prediction performance compared to every single algorithm [16]. This multivariate analysis gave the normalized ensemble importance (ranging from 0 to 1) of each significantly regulated marker found in Fig. 1 (Fig. 3a). The most important markers based on their normalized ensemble importance were thrombocytes (0.86), CD106 (0.72) and Osteopontin (0.63), which also performed well in individual ROC analyses (Fig. 2b). Besides that, EFS was able to pick selected features, which were best suited to discriminate between the two groups. Here, the selected features were thrombocytes, CD106, Osteopontin, CD81, HLA-DR and HBEGF for discrimination between healthy and malaria. To test the performance of the identified feature panel, we performed logistic regression followed by a leave-one out validation of the selected features, which gave an AUC of 0.990 in ROC analysis and a precision and recall (PR)-AUC of 0.9873 for the PR-curve (Fig. 3b, c), supporting the power of the identified feature panel.

**Fig. 4:**
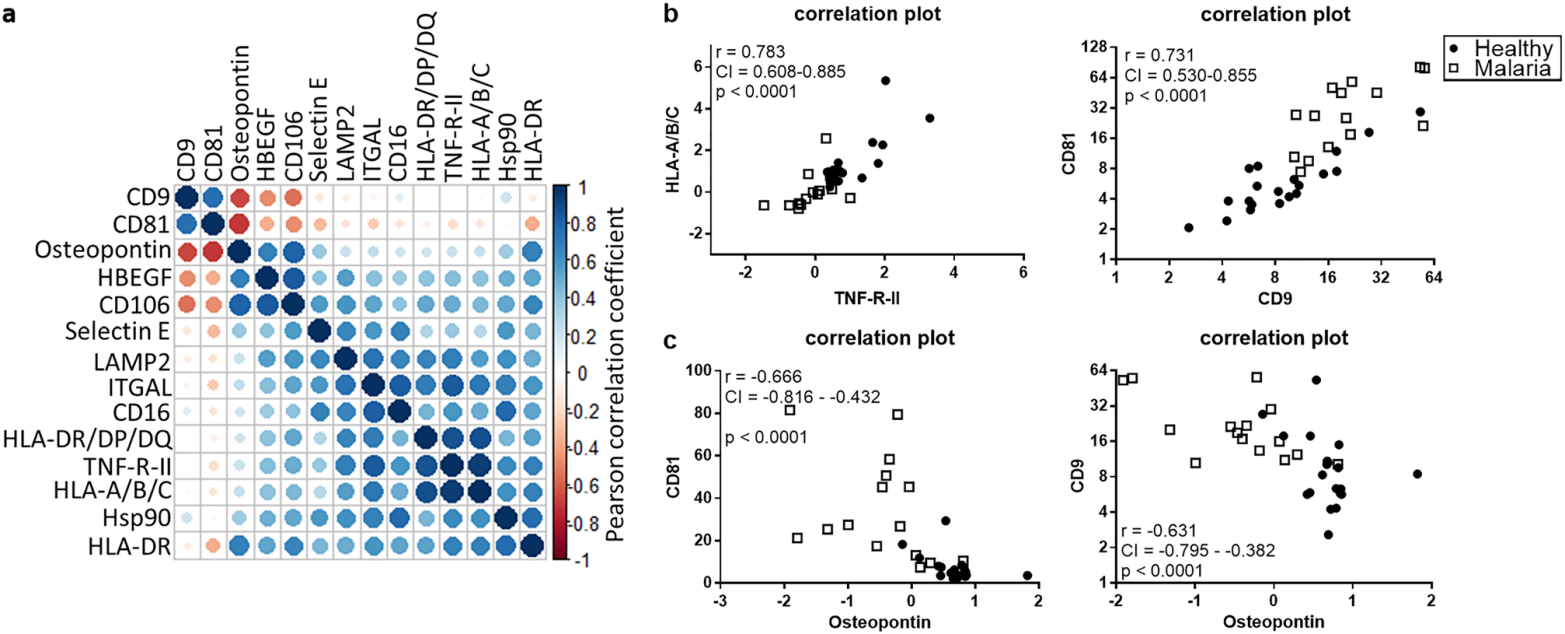
Correlation of sEV proteins. Pearson’s correlation plot of all significantly differentially expressed sEV surface proteins circle size and color indicate correlation (**a**). Correlation plots for the top two positive and negative correlations are shown. TNF-R-II with HLA-A/B/C and CD9 with CD81 (**b**), Osteopontin with CD81 or CD9 (**c**). Correlation coefficient (*r*), 95% confidence interval (CI) and *p* value are depicted in the graphs

To test whether some sEV surface proteins might be expressed simultaneously, we performed Pearson’s correlation analysis among all significantly expressed proteins and found a good overall correlation (Fig. 4a). The best positive correlations were observed for TNF-R-II with HLA-A/B/C (*r* = 0.941) and CD9 with CD81 (*r* = 0.731; Fig. 4b). Negative correlations were observed for osteopontin with CD81 (*r* = − 0.666) or CD9 (*r* = − 0.6314; Fig. 4c). As the thrombocyte concentration did not correlate with any of the significantly regulated sEV proteins, it was excluded from further analyses.

### Markers for malaria severity

As it is critical in clinical routine to distinguish between different severities of malaria, we asked whether the selected feature proteins on sEVs might help for this discrimination. According to World Health Organization (WHO) criteria, severe malaria has been defined as the occurrence of at least one of the subsequent features: impaired consciousness, prostration, multiple convulsions, acidosis, hypoglycemia, severe anemia (hemoglobin < 5 g/dL), acute renal failure (serum creatinine > 3 mg/dL), jaundice, pulmonary edema, significant bleeding, high parasitemia (> 10%) or death [5]. To this end, we used the selected features obtained from EFS analysis (Fig. 3) and separated the patient with severe laboratory alterations (“severe malaria”) from the rest of the malaria group resulting in twelve malaria patients and three severe malaria patients. The malaria patients with severe laboratory alterations were identified based on their high parasite density, LDH, and bilirubin levels and their low thrombocyte counts. There were no statistically significant differences for HLA-DR, Osteopontin, CD81, CD106, and HBEGF between severe malaria and the rest of the malaria cohort (Figure S4a), which might be due to the small group size for the severe malaria patients (*n* = 3), though trends of subtle up- (CD81) or down-regulation (HLA-DR) could be observed. When performing EFS analysis for the discrimination between malaria and the severely ill malaria patients, CD81 (0.59) and CD106 (0.36) were the best markers for discrimination according to their normalized ensemble importance (Figure S4b).

**Fig. 5:**
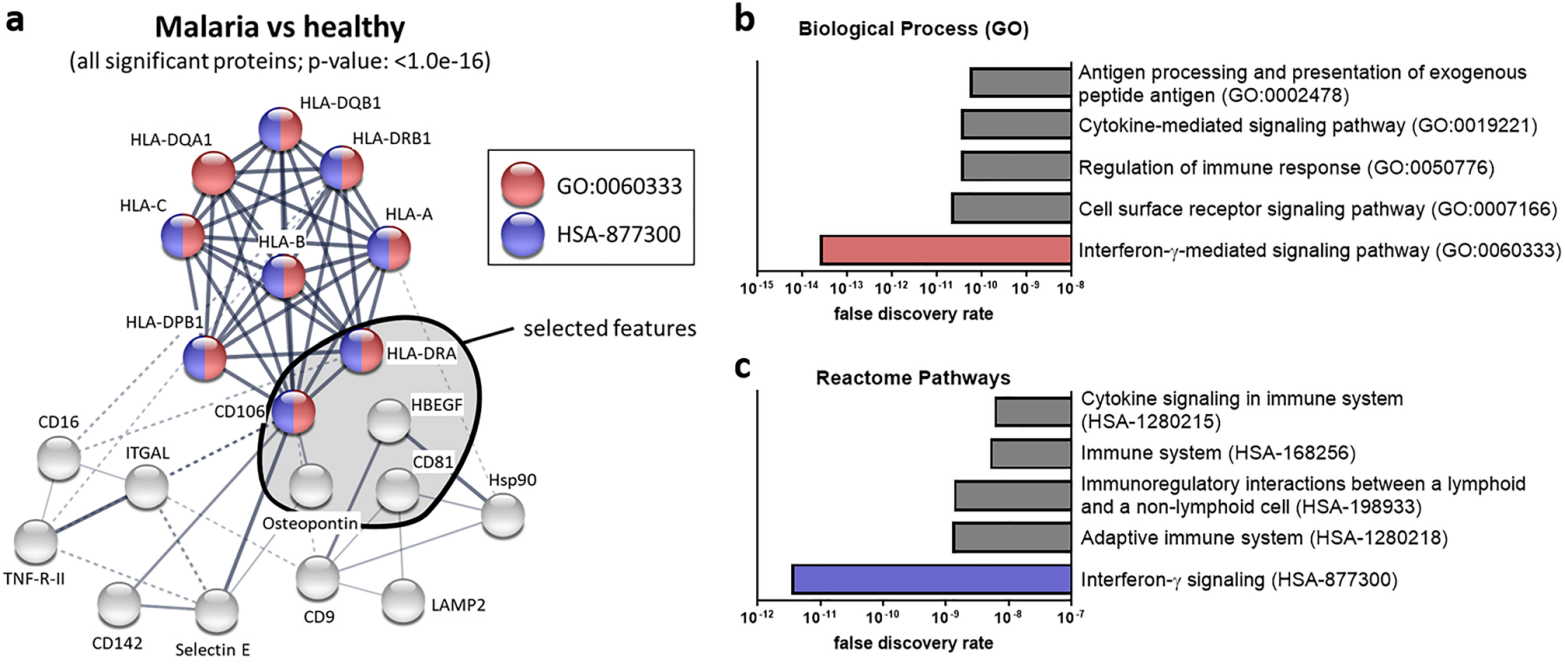
Pathway analysis. **a** A potential protein-protein interaction network was generated with all differentially expressed sEV surface proteins by STRING analysis. Proteins highlighted in red belong to GO:0060333 and proteins highlighted in blue belong to HSA-877300. Selected features for discrimination between healthy and malaria cluster together and are surrounded by the black line. Line thickness indicates the strength of data support. Top five pathways, which might be regulated by differentially expressed sEV surface proteins according to GO-terms (**b**) and reactome (**c**) with their corresponding false discovery rate, are depicted

To address putative interactions of the differentially abundant sEV surface proteins in the plasma of malaria patients, STRING analysis was performed. Proteins were connected with restriction to interactions in humans with all available data sources. The resulting network of proteins (Fig. 5a) interconnects all differentially expressed proteins. The resulting protein network has a protein–protein interaction (PPI) score of *p* < 1.0e−16 compared to a random set of proteins of similar size. The generated PPI network was then tested for possible functions and the top five results for biological processes (GO-terms) and Reactome pathways (HSA) are presented (Fig. 5b, c). The top candidate for function with both databases was by far interferon-γ signaling (GO: 0060333 and HSA-877300). The corresponding proteins are highlighted in the PPI network (red: GO: 0060333, blue: HSA-877300; Fig. 5a).

## Discussion

In this study, we found that the surface proteome of plasma sEVs differs in patients with malaria compared to healthy controls and that thrombocyte levels together with CD106, Osteopontin, CD81, HLA-DR and HBEGF might serve as a feature panel for donor discrimination.

Biomarkers include tools and technologies that can facilitate the prediction, cause, diagnosis, regression or outcome of a treatment of a disease. Moreover, it can help identifying patients at higher risk for acute worsening of the symptoms. Biomarkers for malaria are critical, as the field of malaria research only recently moved in the direction of actively identifying biomarkers that can accurately discriminate the severe forms of malaria [19]. EVs are regarded to be useful biomarkers in diverse medical fields. In clinical routine, it is crucial to implement methods for analysis that only use small volumes of body fluid and that allow to examine a large number of proteins and patient samples at the same time. The EV Array technology used in this study combines these features which heightens the applicability in clinics compared to differential ultracentrifugation, which is to date the most widely used technique for EV isolation [20].

The signal intensities for capturing sEVs with the antibody cocktail against CD9, CD63, and CD81 and detecting them with unspecific IgG antibodies were similar in the two groups, indicating that the total amounts of released sEVs between malaria and healthy controls were comparable. In total, we found 16 proteins to be differentially expressed on plasma sEVs from malaria patients, which were mainly less abundant, compared to healthy controls. The two higher abundant proteins were the tetraspanins CD9 and CD81, which also increased with disease severity. Both, together with CD63, are regarded as exosomal marker proteins. According to the MISEV2018 (minimal information for studies of extracellular vesicles) guidelines [21], they should be present on sEVs but not necessarily in equal amounts. Tetraspanins, as wells as the integrin ITGAL and the lysosome-associated membrane glycoprotein LAMP2, belong to the group of non-tissue specific EV proteins from the MISEV2018 guidelines. In accordance with the MISEV2018 guidelines, these non-tissue specific EV proteins were detected by the EV Array. HLA-DR/DP/DQ are expressed on antigen presenting cells (APCs) and are present in EVs in a cell type or tissue specific manner [21]. The HLA-molecules tested in the EV Array were found to be downregulated in malaria samples compared to healthy controls. Heat shock proteins belong to the group of cytosolic proteins that are recovered in EVs [21] and we observed Hsp90 to be reduced in malaria plasma sEVs. EVs can also transport functional cytokines or growth factors, e.g. EGF [21]. Malaria plasma sEVs transported reduced amounts of HBEGF. Circulating EVs positive for surface HBEGF have been shown to bind to EGFR^+^ endothelial cells, where they promote pro-oxidative and pro-inflammatory responses [22]. As malaria plasma sEVs had reduced levels of HBEGF and EGFR compared to healthy controls, endothelial cell activation might not happen via host sEVs in malaria patients. Yet, it has been published that *Plasmodium* EVs can be taken up by endothelial cells [23] and that the parasite EVs can activate STING in monocytes [24], which makes it likely that these EVs also induce pro-inflammatory signaling in endothelial cells, but we did not study the abundance or function of parasite EVs here. CD106 (also known as V-CAM1) as well as Selectin E (also known as CD62E) are cell adhesion molecules that are mainly found on endothelial cells and their EVs [25], and their reduction on sEVs in malaria patients indicates a lower release of sEVs from endothelial cells. CD142, or tissue factor, which was downregulated here, is a protein that is involved in the clotting process and thrombin formation and CD142^+^ EVs have a pro-coagulant activity [26]. In malaria, coagulation is disturbed by various pathobiological mechanisms including adherence of iRBC to the endothelium, leading to the recruitment and activation of platelets and coagulation [27]. Moreover, the deposition of *Plasmodium* iRBCs occurs by adhesion to the vascular endothelium in the capillary beds of deep tissues [28]. The microvascular endothelium is activated in various disease states (e.g. severe sepsis) by the cytokine TNF-α, triggering the release of endothelial EVs into the bloodstream [29]. Plasma concentrations of TNF-α were shown to be increased in patients with severe malaria and TNF-α levels are discussed to be a potential prognostic biomarker [30]. TNF-α leads to a significant increase in circulating endothelial EVs in malaria patients varying with disease severity. Interestingly, the quantity of these EVs decreases during recovery compared to the acute stage [31]. Endothelial EVs from cell culture experiments have both pro-thrombotic and pro-inflammatory properties [32] and therefore, may contribute to the pathogenesis in severe malaria by promoting fibrin deposition and platelet activation frequently observed during fatal malaria [33]. The complex interplay of TNF-α plasma levels, endothelial-derived EVs and their pathophysiological consequences need to be investigated in the future.

Based on ROC analyses, CD106, CD81, osteopontin, and HLA-DR are best-suited for discrimination between malaria and healthy controls based on their high AUC values (> 0.92). To improve the discrimination, we additionally performed multivariate EFS analysis, which combines eight feature selection algorithms and thereby improves prediction performance compared to each individual analysis [17]. The selected features for the discrimination between healthy and malaria were CD106, CD81, Osteopontin, HLA-DR and HBEGF on plasma sEVs together with the concentration of thrombocytes. Moreover, the sEV surface proteins showed trends for gradual up- (CD81) or down-regulation (HLA-DR) with more severe laboratory alterations in malaria patients.

CD81 is highly expressed on monocyte-derived EVs [34] and was induced in malaria samples. Moreover, the MHC class II molecule HLA-DR was reduced, which is also reduced on monocytes from sepsis patients where it was linked to a reduction in TNF-α response [35]. As malaria plasma samples contained more CD81^+^ sEVs, along with reduced HLA-DR levels, the amount of monocyte-derived EVs might increase during malaria. When monocytes and macrophages receive pro-inflammatory stimuli, they release increased amounts of sEVs [36]. In particular, cells of the monocyte/macrophage lineage are thought to play a key role for host protection against *Plasmodium* in malaria infection, as they phagocytose iRBCs [37] and interact with the parasite itself [38] or with hemolysis products, e.g. extracellular heme [39], leading to downstream cytokine production and modulation of the adaptive immune response. Another bridging cell type of innate and adaptive immunity are dendritic cells (DCs), which are also crucial in malaria infection at every stage of the parasite life cycle [40]. DC-derived EVs are described to be Osteopontin positive [41], which is decreased on plasma sEVs of malaria patients. EVs from activated DCs carry the MHC class I molecules HLA-A/B/C, which in turn can activate resting DCs [42]. The possibility to transfer MHC molecules on EVs can transfer antigen-presenting ability even to T and B cells and they might directly activate T cells [43]. Besides osteopontin and HLA-A/B/C, the cytokine receptor TNF-R-II is also present on DC-derived EVs [42]. CD16, also known as FcγRIII, is expressed on DCs and moreover, CD16^+^ DCs are the only DC subset activated during primary blood-stage human malaria. As they can produce TNF-α as well as IL-10, they can contribute to inflammatory as well as regulatory innate immune processes [44]. CD16 can activate degranulation, phagocytosis and oxidative burst [45]. Its expression on plasma sEVs was reduced in malaria patients. Immature DCs release more EVs than mature DCs [46] and as we observed, reduced levels of TNF-R-II, HLA-class I molecules, Osteopontin and CD16. This argues for a reduced release of sEVs from DCs and more DC activation in malaria patients compared to healthy controls, which is in line with literature [44].

The observed protein pattern on plasma sEVs in malaria patients suggests that immune cells need their receptors for their own activation and do not release them via sEVs. To better understand the potential biological implication of the observed protein composition, pathway analysis by STRING was performed. It revealed a complex PPI network of the differentially expressed proteins and their link to interferon-γ (IFN-γ) signaling. IFN-γ is crucial during *Plasmodium* infection, as it can control parasitemia on the one hand, but on the other hand it can exacerbate its severity by targeting sequestered iRBCs in the brain or the lungs leading to life-threatening complications in severe malaria [47]. This means that the temporal and spatial production of IFN-γ needs to be carefully controlled. As plasma sEVs express proteins that are involved in IFN-γ signaling, the importance of this pathway in malaria is further corroborated.

In future studies, a group with other febrile diseases could be included to assess the difference in sEV surface proteome between different febrile diseases. However, our previous study conducted with plasma samples from community-acquired pneumonia (CAP) patients showed a comparable surface proteome compared to malaria patients for some markers (CD16, CD106, TNF-R-II, Osteopontin, HLA-A/B/C, ITGAL, HLA-DR/DP/DQ, and HBEGF), while others were unique for malaria (CD81, CD9, HLA-DR, Selectin E, Hsp90, LAMP2, EGFR, and CD142) [48]. The shared sEV surface proteome between CAP and malaria argues for a potentially shared profile in acute inflammatory, febrile diseases. Other studies could involve other febrile diseases to further define a core set of markers for acute febrile diseases. Yet, the existence of malaria specific sEV surface proteins suggests a disease-specific regulation of vesicular surface proteins in addition to the general pro-inflammatory phenotype.

Taken together, we characterized the surface proteome of plasma sEVs that allows discrimination between malaria and healthy controls. We cannot conclude yet which effect the herein studied sEVs will have in vivo, as the ultimate function of EVs also depends on the protein and RNA cargo and the recipient cell. For clarification of these questions, future studies are needed.

### Supplementary Information

Below is the link to the electronic supplementary material.

Supplementary file1 (DOCX 1905 KB)

## Data Availability

All data generated or analyzed during this study are included in this article and its supplementary file.

## Abbreviations

AREG: Amphiregulin
AUC_CF: Area under the curve embedded in *cforest*
CI: Confidence interval
CTLA4: Cytotoxic T-lymphocyte associated protein 4
EFS: Ensemble feature selection
EGFR: Epidermal growth factor receptor
ER_CF: Error-rate-based variable importance measure embedded in cf*orest*
ER_RF: Error-rate-based variable importance measure embedded in *randomForest*
Gini_RF: Gini-index-based variable importance measure embedded in *randomForest*
HBEGF: Heparin binding EGF like growth factor
HLA: Major histocompatibility complex
HoxA7: Homeobox A7
Hsp90: Heat shock protein 90
ICAM-1: Intercellular adhesion molecule 1
IFNγ: Interferon-γ
IQR: Interquartile range
iRBC: *Plasmodium*-Infected red blood cell
ITGAL: Integrin subunit alpha L
LAMP2: Lysosomal associated membrane protein 2
LogReg: Logistic regression
MIC-A/B: MHC class I polypeptide-related sequence A/B
PD-L1: Programmed cell death ligand-1
PCA: Principle component analysis
P_cor: Pearson product moment correlation
*P. falciparum*: *Plasmodium falciparum*
PR: Precision and recall
ROC: Receiver operating characteristics
sEV: Small extracellular vesicle
SFTPD: Surfactant protein D
SP-A: Surfactant protein A
TLR3: Toll-like receptor 3
TNF-R: Tumor necrosis factor receptor
Tsg101: Tumor susceptibility gene 101
Tspan8: Tetraspanin 8

## References

[1] WHO. World Malaria Report 2022. 2022. https://www.who.int/teams/global-malaria-programme/reports/world-malaria-report-2022. Accessed 31 Jan 2023.

[2] Rosenthal PJ (2022). Malaria in 2022: challenges and progress. Am J Trop Med Hyg.

[3] Aird WC, Mosnier LO, Fairhurst RM (2014). Plasmodium falciparum picks (on) EPCR. Blood.

[4] English MC (1996). Hyponatraemia and dehydration in severe malaria. Arch Dis Child.

[5] WHO. Guidelines for Malaria. 2022. https://www.who.int/publications/i/item/guidelines-for-malaria. Accessed 31 Jan 2023.

[6] Taylor TE, Borgstein A, Molyneux ME (1993). Acid-base status in paediatric *Plasmodium falciparum* malaria. Q J Med.

[7] Mantel PY, Marti M (2014). The role of extracellular vesicles in Plasmodium and other protozoan parasites. Cell Microbiol.

[8] Yanez-Mo M (2015). Biological properties of extracellular vesicles and their physiological functions. J Extracell Vesicles.

[9] van Niel G, D'Angelo G, Raposo G (2018). Shedding light on the cell biology of extracellular vesicles. Nat Rev Mol Cell Biol.

[10] Mantel PY (2013). Malaria-infected erythrocyte-derived microvesicles mediate cellular communication within the parasite population and with the host immune system. Cell Host Microbe.

[11] Couper KN (2010). Parasite-derived plasma microparticles contribute significantly to malaria infection-induced inflammation through potent macrophage stimulation. PLoS Pathog.

[12] Xu R (2016). Extracellular vesicle isolation and characterization: toward clinical application. J Clin Investig.

[13] Jung AL (2019). Surface proteome of plasma extracellular vesicles as biomarkers for pneumonia and acute exacerbation of chronic obstructive pulmonary disease. J Infect Dis.

[14] Jorgensen MM, Baek R, Varming K (2015). Potentials and capabilities of the extracellular vesicle (EV) array. J Extracell Vesicles.

[15] Jorgensen M (2013). Extracellular vesicle (EV) array: microarray capturing of exosomes and other extracellular vesicles for multiplexed phenotyping. J Extracell Vesicles.

[16] Neumann U, Genze N, Heider D (2017). EFS: an ensemble feature selection tool implemented as R-package and web-application. BioData Min.

[17] Neumann U (2016). Compensation of feature selection biases accompanied with improved predictive performance for binary classification by using a novel ensemble feature selection approach. BioData Min.

[18] Gebreweld A (2021). Thrombocytopenia as a diagnostic marker for malaria in patients with acute febrile illness. J Trop Med.

[19] Sahu PK (2015). Pathogenesis of cerebral malaria: new diagnostic tools, biomarkers, and therapeutic approaches. Front Cell Infect Microbiol.

[20] Sandfeld-Paulsen B (2016). Exosomal proteins as prognostic biomarkers in non-small cell lung cancer. Mol Oncol.

[21] Thery C (2018). Minimal information for studies of extracellular vesicles 2018 (MISEV2018): a position statement of the International Society for Extracellular Vesicles and update of the MISEV2014 guidelines. J Extracell Vesicles.

[22] Burger D (2011). Endothelial microparticle formation by angiotensin II is mediated via Ang II receptor type I/NADPH oxidase/Rho kinase pathways targeted to lipid rafts. Arterioscler Thromb Vasc Biol.

[23] Babatunde KA (2018). Malaria infected red blood cells release small regulatory RNAs through extracellular vesicles. Sci Rep.

[24] Sisquella X (2017). Malaria parasite DNA-harbouring vesicles activate cytosolic immune sensors. Nat Commun.

[25] Jung KH (2009). Circulating endothelial microparticles as a marker of cerebrovascular disease. Ann Neurol.

[26] Li M (2010). Tobacco smoke induces the generation of procoagulant microvesicles from human monocytes/macrophages. Arterioscler Thromb Vasc Biol.

[27] Ghosh K, Shetty S (2008). Blood coagulation in falciparum malaria—a review. Parasitol Res.

[28] Lou J, Lucas R, Grau GE (2001). Pathogenesis of cerebral malaria: recent experimental data and possible applications for humans. Clin Microbiol Rev.

[29] Elsner C, Ergun S, Wagner N (2023). Biogenesis and release of endothelial extracellular vesicles: morphological aspects. Ann Anat.

[30] Mahittikorn A (2022). Tumour necrosis factor-alpha as a prognostic biomarker of severe malaria: a systematic review and meta-analysis. J Travel Med.

[31] Combes V (2004). Circulating endothelial microparticles in malawian children with severe falciparum malaria complicated with coma. JAMA.

[32] Combes V (1999). In vitro generation of endothelial microparticles and possible prothrombotic activity in patients with lupus anticoagulant. J Clin Investig.

[33] Francischetti IM, Seydel KB, Monteiro RQ (2008). blood coagulation, inflammation, and malaria. Microcirculation.

[34] Pugholm LH (2016). Phenotyping of leukocytes and leukocyte-derived extracellular vesicles. J Immunol Res.

[35] Winkler MS (2017). Human leucocyte antigen (HLA-DR) gene expression is reduced in sepsis and correlates with impaired TNFalpha response: a diagnostic tool for immunosuppression?. PLoS One.

[36] Jung AL (2017). Legionella pneumophila infection activates bystander cells differentially by bacterial and host cell vesicles. Sci Rep.

[37] Chua CL (2013). Monocytes and macrophages in malaria: protection or pathology?. Trends Parasitol.

[38] Krishnegowda G (2005). Induction of proinflammatory responses in macrophages by the glycosylphosphatidylinositols of *Plasmodium falciparum*: cell signaling receptors, glycosylphosphatidylinositol (GPI) structural requirement, and regulation of GPI activity. J Biol Chem.

[39] Figueiredo RT (2007). Characterization of heme as activator of Toll-like receptor 4. J Biol Chem.

[40] Yap XZ (2019). Dendritic cell responses and function in malaria. Front Immunol.

[41] Silva AM (2017). Dendritic cell-derived extracellular vesicles mediate mesenchymal stem/stromal cell recruitment. Sci Rep.

[42] Obregon C (2009). Active uptake of dendritic cell-derived exovesicles by epithelial cells induces the release of inflammatory mediators through a TNF-alpha-mediated pathway. Am J Pathol.

[43] Andre F (2004). Exosomes as potent cell-free peptide-based vaccine. I. Dendritic cell-derived exosomes transfer functional MHC class I/peptide complexes to dendritic cells. J Immunol.

[44] Loughland JR (2019). *Plasmodium falciparum* activates CD16+ dendritic cells to produce tumor necrosis factor and interleukin-10 in subpatent malaria. J Infect Dis.

[45] Murphy K, Weaver C (2001). Janeway's immunobiology, vol 9.

[46] Segura E (2005). ICAM-1 on exosomes from mature dendritic cells is critical for efficient naive T-cell priming. Blood.

[47] King T, Lamb T (2015). Interferon-gamma: the Jekyll and hyde of malaria. PLoS Pathog.

[48] Jung AL (2020). Surface proteome of plasma extracellular vesicles as biomarkers for pneumonia and acute exacerbation of chronic obstructive pulmonary disease. J Infect Dis.

