# Supplementary material for: Surface proteome of plasma extracellular vesicles as mechanistic and clinical biomarkers for malaria
Source: Infection. 2023 Mar 24;51(5):1491–501. doi: 10.1007/s15010-023-02022-x (PMC10545645; doi:10.1007/s15010-023-02022-x)
Supplement: Supplementary file 1 — Supplementary file1 (DOCX 1905 KB) [file 15010_2023_2022_MOESM1_ESM.docx]

**Supplementary Material**

**Figure S1:**





**Figure S1:** a/c) Laboratory blood test values. Data is presented as Box-Whisker plots. Line is showing the median, boxes are showing 25^th^ to 75^th^ percentile of the data, Whiskers are presenting the 1.5IQR (inter-quartile range; Tukey) and outliers. a) Healthy and malaria; c) malaria only. b) ROC of thrombocyte. Receiver operating characteristics (ROC) analysis for discrimination between healthy and malaria for thrombocytes/nL is shown. Area under the curve (AUC), 95% confidence interval (CI) and p-value are depicted in the graph. d) EV Array signal intensities for biotinylated IgG antibody. Values were log2-transformed and are presented in Box-Whisker plots. Line is showing the median, boxes are showing 25^th^ and 75^th^ percentile of the data with the 1.5IQR (inter-quartile range; Tukey). Statistics: unpaired t-test with Welch’s correction; * in comparison to healthy; **** p<0.0001, ** p<0.01, * p<0.05; ns = not significant.

**Figure S2:**





**Figure S2: Differential abundance of sEV surface proteins.** EV Array signal intensities for all not-significantly differentially expressed sEV surface proteins. Values were log2-transformed and are presented in Box-Whisker plots. Line is showing the median, boxes are showing 25^th^ to 75^th^ percentile of the data with Whiskers presenting the 1.5IQR (inter-quartile range; Tukey) and outliers. Statistics: unpaired t-test with Welch’s correction.

**Figure S3:**

**

**

**Figure S3: Differential abundance of sEV proteins based on gender.** a/b) EV Array signal intensities for all significantly differentially expressed sEV surface proteins between male and female (a) HBEGF; b) Osteopontin). Values were log2-transformed and are presented in Box-Whisker plots. Line is showing the median, boxes are showing 25^th^ to 75^th^ percentile of the data with Whiskers presenting the 1.5IQR (inter-quartile range; Tukey) and outliers. Statistics: unpaired t-test with Welch’s correction; ** p<0.01, * p<0.05; ns = not significant.

**Figure S4:**





**Figure S4: Malaria severity markers. a**) EV Array signal intensities for selected features (HLA-DR, Osteopontin, CD81, CD106 and HBEGF) for discrimination between healthy, malaria and malaria patients with severe laboratory alterations (“severe malaria”). Values were log2-transformed and are presented in Box-Whisker plots. Line is showing the median, boxes are showing 25^th^ to 75^th^ percentile of the data with Whiskers presenting the 1.5IQR (inter-quartile range; Tukey) and outliers. b) Cumulative barplot of individual features for all feature selection methods is shown. P_cor: Pearson product moment correlation, LogReg: logistic regression, ER_RF: error-rate-based variable importance measure embedded in *randomForest*, Gini_RF: Gini-index-based variable importance measure embedded in *randomForest*. Statistics: unpaired t-test with Welch’s correction; * in comparison to healthy; **** p<0.0001, *** p<0.001, ** p<0.01, * p<0.05.
